# Natural Antioxidant Application on Fat Accumulation: Preclinical Evidence

**DOI:** 10.3390/antiox10060858

**Published:** 2021-05-27

**Authors:** Proshanta Roy, Daniele Tomassoni, Enea Traini, Ilenia Martinelli, Maria Vittoria Micioni Di Bonaventura, Carlo Cifani, Francesco Amenta, Seyed Khosrow Tayebati

**Affiliations:** 1 School of Biosciences and Veterinary Medicine, University of Camerino, 62032 Camerino, Italy; proshanta.roy@unicam.it (P.R.); daniele.tomassoni@unicam.it (D.T.); 2 School of Pharmacy, University of Camerino, 62032 Camerino, Italy; enea.traini@unicam.it (E.T.); ilenia.martinelli@unicam.it (I.M.); mariavittoria.micioni@unicam.it (M.V.M.D.B.); carlo.cifani@unicam.it (C.C.); francesco.amenta@unicam.it (F.A.)

**Keywords:** antioxidant, obesity, inflammation, preclinical studies

## Abstract

Obesity represents one of the most important challenges in the contemporary world that must be overcome. Different pathological consequences of these physical conditions have been studied for more than 30 years. The most nagging effects were found early in the cardiovascular system. However, later, its negative impact was also investigated in several other organs. Damage at cellular structures due to overexpression of reactive oxygen species together with mechanisms that cause under-production of antioxidants leads to the development of obesity-related complications. In this view, the negative results of oxidant molecules due to obesity were studied in various districts of the body. In the last ten years, scientific literature has reported reasonable evidence regarding natural and synthetic compounds’ supplementation, which showed benefits in reducing oxidative stress and inflammatory processes in animal models of obesity. This article attempts to clarify the role of oxidative stress due to obesity and the opposing role of antioxidants to counter it, reported in preclinical studies. This analysis aims to clear-up different mechanisms that lead to the build-up of pro-oxidants during obesity and how various molecules of different origins hinder this phenomenon, behaving as antioxidants.

## 1. Introduction

In the last years, several studies tried to spot the link between antioxidant compounds and oxidative stress due to obesity. Obesity is one of the public health problems because it may lead to metabolic and cardiovascular disorders. Obesity is a chronic and multifactorial disease characterized by an increase in the white adipose tissue (WAT) that results mainly in fat accumulation localized in the abdominal region [1]. Obesity is considered a medical challenge because it is associated with chronic disease development. Actually, high body mass index (BMI) or the increase of waist circumference is also correlated with the development of cardiovascular risk factors such as hypertension, dyslipidemia, insulin resistance, and diabetes mellitus [2,3,4,5]. In a meta-analysis, including 2.88 million individuals, all obesity grades were associated with a significant increase in mortality rate [6].

**Figure 1:**
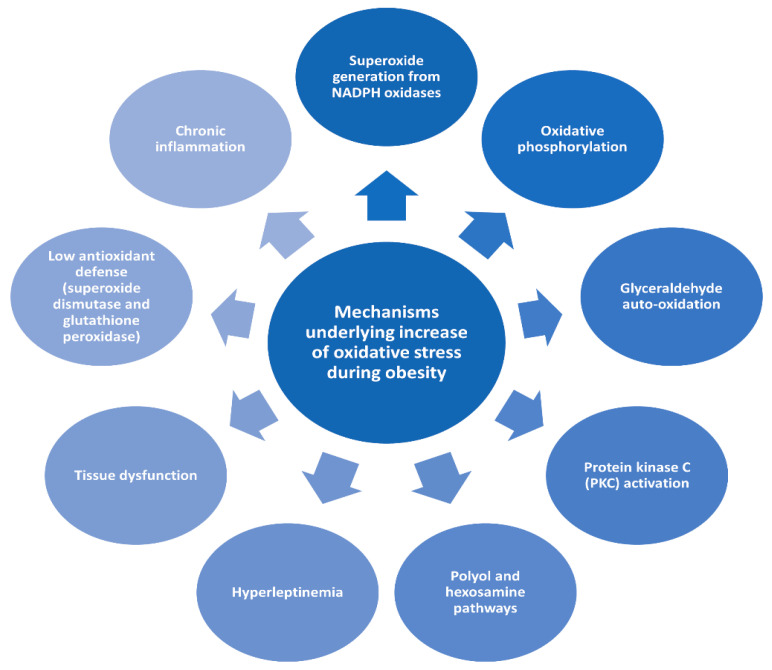
Mechanisms underlying increase of oxidative stress during obesity.

Oxidative stress plays an essential role in the development of co-morbidities in obese condition [7]. Obesity promotes oxidative stress through different biochemical mechanisms [7], as summarized in Figure 1. Moreover, evidence accumulated over the past two decades has pointed to significant connections between inflammation and oxidative stress, where every process contributes to fuel the other one, thereby establishing a vicious cycle able to perpetuate and propagate the inflammatory response. This correlation is mediated by the downregulation of nuclear factor kappa-light-chain-enhancer of activated B cells (NF-κB) signaling, the activation of the NLR family pyrin domain containing 3 (NLRP3) inflammasome, and Toll-like receptors (TLR). On the TLR, the extracellular domain connects through a transmembrane domain to the cytoplasmic Toll/interleukin-1 receptor (TIR) domain, which interacts with adaptor molecules. Consequently, the expression of inflammatory mediators is upregulated, comprising notably pro-oxidant enzymes such as NADPH oxidase (NOX) and inducible nitric oxide synthase (iNOS) and producing high levels of reactive oxygen species (ROS). TLR engagement also facilitates the generation of ROS within mitochondria and promotes activation of NOX [8].

The role of plant-derived antioxidants in food and human health has been widely investigated. The positive effect of many food products and beverages such as fruits, vegetables, coffee, tea, and cacao on human health is due to their antioxidant activity [9]. Antioxidants’ supplementation is not necessary in a balanced diet. Indeed, the Mediterranean diet, characterized more by intake of plant-based foods and fish and less consumption of meat and dairy products, represents the gold standard in preventive medicine, thanks to the synergy of many foods with antioxidant and anti-inflammatory effects [10]. As reviewed in [11], clinical trials failed to demonstrate beneficial effects of synthetic antioxidants’ supplementation in preventing diseases. There is also some concern that inappropriate use of dietary supplements may induce “antioxidative stress” and thus they may be harmful. Before prescribing antioxidants, an accurate determination of an individual’s oxidative stress levels is required [11].

Antioxidants are compounds that decrease the levels of ROS, which can modulate mechanisms of the homeostasis of glucose, lipids, and amino acids and suppress inflammation [12,13,14]. 

The development of functional foods for health benefit is important to the determination of bioaccessibility and bioavailability [15]. Having various compounds in bioactive food molecules that result in a complex chemical structure by adding the lipophilic and hydrophilic molecules may differ the absorption mechanisms. There is also a significant difference in absorption of polyphenols groups, even within the same subclass of compounds [16]. Phenolic compounds are cited as a secondary metabolite in plants. At low concentration, phenolic compounds may act as an antioxidant. On the contrary, at high concentrations, these compounds may interact with proteins, carbohydrates, and minerals [17]. Flavonoids are polyphenolic antioxidant compounds that are typically both ROS scavengers and metal chelators that potentially produce double protection. They are generally accepted as heavily advocated antioxidants for the interesting polymer-based delivery and its short-time circulation ability, which correlate their antioxidant activity with the degree of polymerization [18]. 

Sugar molecules have an effective role in the absorption of phenolic compounds. If the phenolic compounds contain a sugar molecule, such as galactose or xylose, glucose, they will be absorbed through the small intestine by the cytosolic β-glucosidase/lactase phlorizin hydrolase [19]. If the sugar molecules are not present, the hydrophilic cannot be absorbed in the upper gastrointestinal part. (−)-epicatechin (EC) and (−)-epigallocatechin (EGC) are acylated flavonoids. They can be directly absorbed without deconjugation and hydrolysis [20]. The aglycone is a phase II substrate for metabolism, typically glucuronidation, sulfation, and methylation, carried out by UDP-glucuronosyltransferases, sulfotransferases, and catechol-*O*-methyltransferases (COMT) [21]. During the phase II of metabolism, metabolites are rapidly reached in the liver via the portal vein.

In vitro methods result in the changes in bioactive compounds’ bioaccessibility with the variations of food matrix and food processing. Several studies have demonstrated that the bioavailability and bioactivity of some functional components receive support from microbiota. Increasing the antioxidant bioactivity using probiotics and prebiotic strains is an innovative manner, which alternatively increases the involvement of natural and metabolic components [22]. The rearrangement of the structure of polyphenols (by the addition or removal of hydroxyl and methoxy groups) at the colonic level might affect metabolism on microbiota [23].

Increasing attention has been given to the different strategies and possible therapies targeting differentiation of adipogenesis, glucose intake and transport, attenuation of inflammation, and changes within the immune response. Long-term and low-level inflammation usually present in obese subjects, and alterations presented within the metabolism, could lead to changed immunity. Understanding the mechanisms of action of antioxidants in human health conservation and disease prevention could promote interest in new drug discovery. Besides, it may also clear the potential of diets in the prevention of obesity and several diseases linked to it. This review attempts to clarify the role of natural antioxidant compounds in oxidative stress and inflammation due to obesity, focusing on preclinical studies.

## 2. Natural Antioxidant Compounds

### 2.1. Flavonoids

More and more people are coming to discover the powerful healing properties of antioxidants. One of them that is getting the most attention is anthocyanin.

Anthocyanin represents one of the largest groups of phenolic pigments with antioxidant properties. The molecules are found in red wine, some cereals, root vegetables, and red and purple fruits. Anthocyanins are potentially integrated into food and in medical products by pharmaceutical industries due to their potential health benefits [24]. They are backed up by new research that shows their beneficial effects. Anthocyanin has a high antioxidant potential as measured by laboratory tests and contains several compounds that fight free radicals [25,26]. They have been shown to cut down on inflammation of different sorts, including decreased muscle soreness, greater strength following exercise, and decreased inflammatory markers [27,28,29]. Anthocyanin has beneficial metabolic effects, such as decreasing fat, sugar, and insulin levels in the blood [30].

It showed useful effects on sleep, encouraging positive effects against some cancers such as reducing tumor burden in the gut and slowing tumor growth, and it might promote brain health [31,32,33]. Anthocyanins are antioxidants that eliminate ROS. In particular, among them, delphinidin represents the most active scavenger against superoxide anions. In cell lines, a different study reports that anthocyanin has protective effects against oxidative stress [34]. In vivo studies demonstrated the antioxidant functions of anthocyanins. Cyanidin-3-glucoside improved oxidative stress-induced hepatic ischemia-reperfusion in rats, and cyanidin, delphinidin, and malvidin induced upregulation of antioxidant response element (ARE) pathways. Moreover, anthocyanins are effective against cytotoxicity, lipidic peroxidation, and as protectors of DNA. Besides, the capacity of the anthocyanins for stabilizing triple-helical complexes of DNA by forming complexes of anthocyanins-DNA is well-established [24]. Anthocyanins have cellular antioxidant mechanisms comparable to or greater than other micronutrients, such as vitamin E. Indirectly, anthocyanins show anti-inflammatory effects: cyanidin-3-glucoside, delphinidin-3-glucoside, and petunidin-3-glucoside inhibited NF-κB activities, while other cyanidins inhibited cyclooxygenase enzyme activities [34].

**Figure 2:**
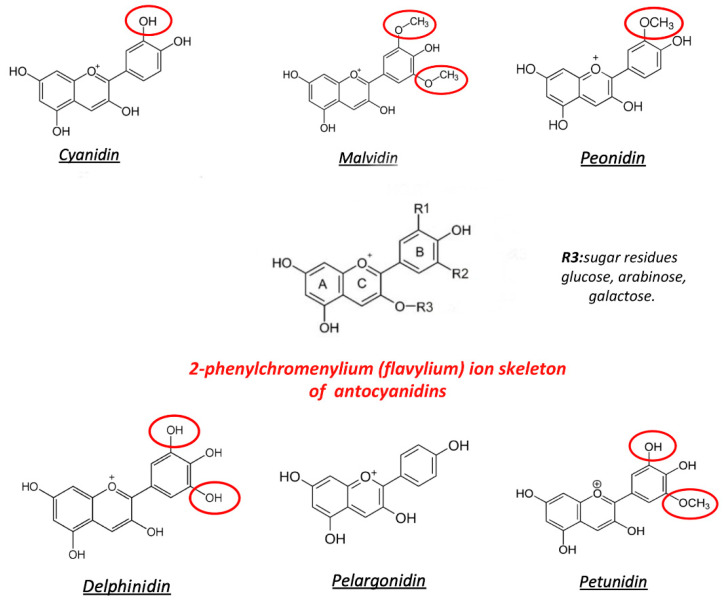
Structure of the most common anthocyanins. Red circles represent the substitutions of the flavylium group, in the positions R1 and R2.

Basically, anthocyanin is a subfamily of flavonoids (Figure 2), which are abundant in fruits, seeds, and plant leaves [35]. Six major compounds are derived from the basic structure of 2-phenylchromenylium (flavylium B-ring). They are cyanidin, delphinidin, malvidin, pelargonidin, peonidin, and petunidin, depending on their flavylium B-ring substitutions. Sugars such as glucose, arabinose, and galactose can be attached to the basic structure of anthocyanins [36,37] and could modulate the changes in the antioxidant activity. [38]. For example, in cyanidin, glycosylation in position 3 of the flavylium B-ring with glucose or rhamnose increases the antioxidant activity, but with galactose, it declines, as shown by Oxygen Radical Absorbance Capacity (ORAC) assays [24].

The scientific literature on anthocyanin concerns small changes in the instance of the amount and position of oxygen–hydrogen (hydroxyl) units on the caffeic acid structures, which act like cannons on a ship ready to aim free radicals. Besides, when the smoke cleared, these compounds turned out to be nearly as effective as other commercial antioxidants, namely BHT (butylated hydroxytoluene) and TBHQ (tert-butylhydroquinone). Furthermore, the anthocyanin compounds may work in an additional way, that is, they may chelate or build-up metals (e.g., iron) in the body, which produce free radicals such as hydroxyls that cause oxidative stress and organ damage [39].

Evidence continues to mount for the anti-inflammatory and antioxidant effects of anthocyanin. Different researchers checked out how cherries blocked lipid peroxidation of the plasma membrane. All of the compounds, including the base structure of cyanidin (without sugar attached), and, therefore, the three major sugar-added compounds, blocked this peroxidation. Even certain glycoside products of cyanidin appear to hold onto iron and other heavy metals, which may cause peroxidation. Overall, cyanidin actually performed better than aspirin at inhibiting cyclooxygenase (COX) [40].

Anthocyanins have the power to eliminate ROS. In vitro, a large number of studies have proven that anthocyanins, in particular cyanidin-3-glucoside, have an ORAC [41]. Delphinidin is the most active scavenger against superoxide anion [42]. Moreover, various studies suggest that it has a protective effect against oxidative stress in different cell lines [43,44,45]. In vivo, the antioxidant effect of anthocyanin is positive. The upregulation of the ARE pathways is induced by cyanidin, delphinidin, and malvidin antioxidant enzymes. [46].

Studies highlighted that the consumption of anthocyanins reduced body weight and insulin resistance, resulting in restored glucose tolerance. Mitogen-activated protein kinase (MAPK) pathways induce cyanidin-3-glucoside, delphinidin-3-glucoside, and petunidin-3-glucoside to inhibit NF-κB [47,48,49], and also the activities of cyclooxygenase enzyme were inhibited by cyanidins [50]. These biological activities of anthocyanins are closely associated with the kind and progression of diseases [24].

Red cabbage microgreen, blueberry, blackcurrant, mulberry, cherry, black elderberry, black soybean, chokeberry, and jaboticaba’s peel contain a variety of anthocyanins, including cyanidins, delphinidins, malvidins, pelargonidins, peonidins, and petunidins, that showed a different positive effect on an animal model of obesity. Red cabbage microgreen decreased weight gain, low-density lipoprotein (LDL) levels, triacylglycerol, and cholesterol levels in high-fat diet-fed mice. Inflammatory cytokines such as C-reactive protein (CRP) and tumor necrosis factor alpha (TNF-α) also significantly decreased in mice. In high-fat diet (HFD) mice, the supplementation with blueberry has shown a reduction in body weight and blood glucose levels as well as TNF-α and interleukin 6 (IL-6) levels. The whole blueberry improved high-fat diet-induced insulin resistance and decreased TNF-α, IL-6, monocyte chemoattractant protein-1 (MCP-1), CD11c^+^ also known as integrin alpha X, and iNOS. Again, in HFD mice, blueberry juice reduced the weight gain, the plasma level of insulin and leptin, the cholesterol, and triacylglycerol in the liver, reducing inflammatory markers such as TNF-α, IL-6, iNOS, and NF-kB in WAT. A similar effect was highlighted in HFD mice supplemented with anthocyanin-rich fruit such as blackcurrant, mulberry, cherry, black elderberry, black soybean, and freeze-dried jaboticaba peel. Chokeberry extracts led to improve metabolic disturbance and inflammation in rats with a fructose-rich diet, while tart cherry reduced metabolic and inflammatory markers in Zucker fatty rats [34].

Recently, the supplementation of tart cherry juice and seeds rich in anthocyanins, even if it did not reduce the weight gain, was able to reduce the neuroinflammatory process, liver steatosis, inflammation, and adipose gene transcription in visceral adipose tissue in rats under diet-induced obesity (DIO) [51,52,53,54]. Several analyses find that some compounds showed higher antioxidant activities than other compounds, particularly cyanidin derivatives such as kaempferol, quercetin, and melatonin. Each of these compounds could contribute to the antioxidant effects attributed to crude extracts of tart cherry fruit. However, some minimal level of particular constituents is needed for these molecules to exhibit their antioxidant effects most efficiently. Concentrations of antioxidant compounds in cherry may be influenced by many factors, including environmental conditions, degree of ripeness, cultivar, cultivation site, processing, and storage of the fruit [50].

The anthocyanins and other flavonoids, similarly to melatonin, found in tart cherry fruits are reported to have various phytotherapeutic activities that are supported by their modes of action at different target sites [55].

Quercetin has been shown to inhibit COX and lipoxygenase activities. These enzymes are involved in the release of arachidonic acid, the initiator of a general inflammatory response. Quercetin also exerts a preferential cytotoxic effect on dividing colon carcinoma HT29 and CACO-2 cells and induces apoptosis in human leukemia HL60 cells, following inhibition of growth. Possible mechanisms of action could include increased expression of wild-type p53, reduction of Ki RAS levels, or p21 upregulation. Besides, animal studies indicate that the incidence of carcinogen-induced mammary tumors and lung tumors was decreased by dietary administration of quercetin [56]. The flavonols, quercetin, and catechin synergistically act to inhibit platelet aggregation and adhesion to collagen, reducing atherosclerotic lesions [57]. The antioxidant effectiveness of anthocyanins and other polyphenols, in vitro, is essential because of the spontaneity of the chemical structure in which a hydrogen atom from an aromatic hydroxyl group is donated to a free radical. Besides, their ability to chelate transition metal ions, involved in radical forming processes such as Fenton reactions and the induction of endogenous antioxidants, could also contribute to the antioxidant efficacy of these compounds. Consequently, numerous studies have shown that many phenolic compounds found in fruits and vegetables, including berries, inhibit the oxidation of LDL and DNA in vitro [58].

Membrane lipid viscosity or protein movement is probably affected by the flavonoids. However, the combined bioactive mixture is potentiated when the interaction leads to improved solubility, absorption, safety, stability, or bioavailability of the active principles. Such synergistic interactions are also explained by enhanced uptake, absorption, metabolism, and reduced excretion (pharmacokinetics), or by enhanced effectiveness (binding to receptor molecules like bioactive proteins that enhance protein–protein or protein–ligand interactions) at the target sites of action (pharmacodynamics). This idea has been adopted by pharmacologists to explore combinations of several metabolites in multi-target therapy. The importance of affecting multiple targets may be beneficial when handling complex diseases, such as cancer, chronic inflammation, chronic infection, and plenty of others [59].

### 2.2. Resveratrol

**Figure 3:**
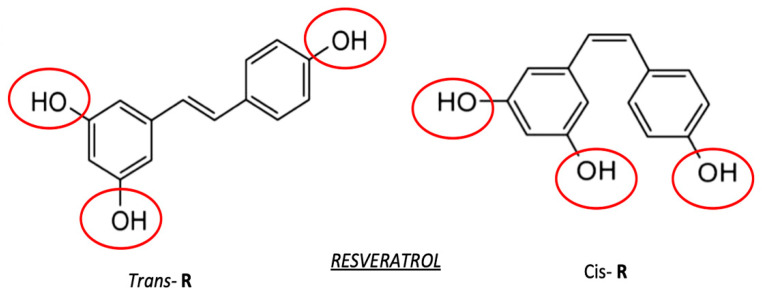
Configuration of *trans* (left part) and *cis* (right part) forms of RSV. Red circles represent the reactive site of the molecule.

Resveratrol (trans-3, 4′, 5-trihydroxystilbene, RSV, Figure 3) is a small polyphenol that has been largely studied for decades in a wide spectrum of therapeutic research areas [60,61]. The natural occurrence of RSV in a large variety of plant species, specifically, mulberries, peanuts, and grapes, has further fostered public opinion and claims around the possibility of using RSV within the fields of natural medicine and dietary supplementations [62].

In obese subjects, RSV exhibited a vascular protective effect, mimicking calorie restriction [63,64,65]. RSV also has an antioxidant effect, protected from deregulated glucose tolerance that leads to a beneficial effect on hypertriglyceridemia and improves memory performance (maintenance of brain health) [63].

Supplementation with RSV of rodents protected animals against HFD-induced body weight gain and obesity. RSV also increased the energy expenditure, which was partly mediated by stimulating intracellular mitochondrial functions (fatty acid oxidation) in adipose tissue, by the fatty acid synthesis suppression, and by producing brown-like adipocyte formation in WAT [66,67,68,69,70,71,72]. The in vitro anti-inflammatory effect of RSV was also confirmed in animal models. In mice, RSV reduced HFD-induced inflammation of WAT by downregulating pro-inflammatory cytokines TNF-α, interferon alpha and beta (IFN-α and IFN-β), and IL-6 [60,73]. Moreover, RSV prevented the production of regulatory T cells and reduced adipose tissue macrophage infiltration in HFD-induced obese mice [74]. In Zucker rats, the levels of IL-6 and the activity of NF-κB were suppressed by RSV reducing macrophage infiltration in adipose tissue [60]. Amusingly, Jimenez-Gomez et al. showed that RSV reported similar effects on a high-fat-treated adult rhesus monkey model as effects on HFD-induced obese rodent models. RSV decreased the mRNA levels, suppressed the activation of pro-inflammatory cytokines IL-6, TNF-α, IL-1β, and NF-κB, and adiponectin in the visceral adipose tissue of the HFD-treated monkey model [75]. The antioxidant effect of RSV was also proven in other animal models. Lv and co-workers reported that RSV reduced oxidative stress, which is associated with diet induced partly by the reduction of sirtuin 1 (SIRT1) and manganese superoxide dismutase (MnSOD) levels [76].

Obesity is induced by providing a high-calorie diet (e.g., excessive amount of dietary fat or sugar). Common markers for obesity include weight and fasting serum levels of glucose and insulin. The effect of RSV on body weight is controversial, but it is demonstrated that there is a significant difference in glucose tolerance between standard diet and fat/sugar-enriched diet controls [62]. It was also demonstrated that RSV regimens improved glucose tolerance or lowered fasting glucose level and decreased serum insulin, in mice or swine models, compared with obese controls [77].

Numerous studies have reported the anti-inflammatory properties of RSV in several inflammation models, including arthritis, asthma, encephalomyelitis, atherosclerosis, and intestinal inflammatory diseases, among others [78,79,80,81]. Nevertheless, recently, the effect of RSV on the inflammatory process, which takes place in adipocytes under several metabolic conditions such as obesity, was studied.

The vast majority of these recent studies have been performed in in vitro conditions, by using cultured adipocytes or macrophages [67,82,83], or adipose tissue explants [84]. These data demonstrate that RSV may prevent inflammatory processes in adipocytes, but it is essential to remember that important limitations exist when extrapolating these data to the in vivo situation.

**Figure 4:**
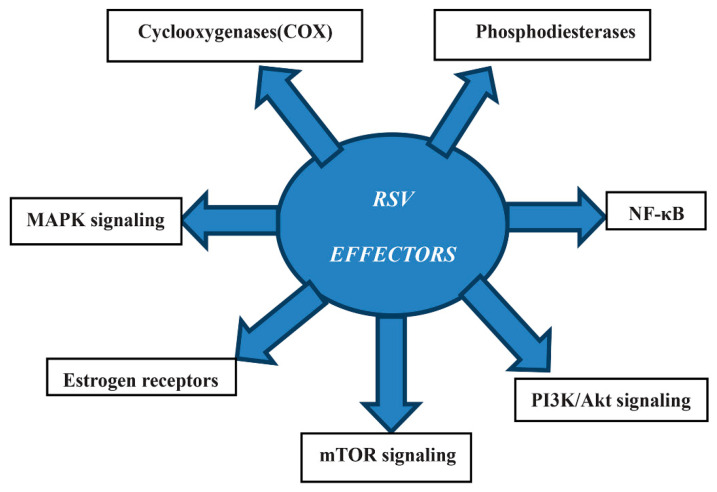
Mechanism of action of RSV effectors. Abbreviations: nuclear factor kappa-light-chain-enhancer of activated B cells (NF-κB), phosphatidylinositol 3-kinase (PI3K)/protein kinase B (AKT), mechanistic target of rapamycin (mTOR), mitogen-activated protein kinase (MAPK).

Various studies have shown the in vivo anti-inflammatory effects of several polyphenols, such as quercetin [85] or polyphenol extracts [86,87,88]. There are some other possible effectors which are additionally relevant targets proposed for RSV (Figure 4). Particularly, investigations on classic and novel possible targets relevant for the metabolic effects of RSV, and, more specifically, the direct action of RSV, have been described [89,90,91,92].

Despite that RSV has interesting properties and potential applications, there are some limits for its use in aqueous formulations, due to its poor solubility, stability, and bioavailability. To overcome these limitations, various solutions are being studied, including the development of new formulations using nanoparticles or nanoemulsions.

### 2.3. Thioctic Acid

Thioctic acid (TIO), also called alpha-lipoic acid, is a naturally occurring short-chain fatty acid that contains a thiol bond. It is an essential cofactor for energy production in the mitochondria [93]. TIO also has a powerful antioxidant effect, and it is a free radical scavenger. TIO is marketed in several places as an over-the-counter nutritional antioxidant supplement, alone or together with other antioxidants.

**Figure 5:**
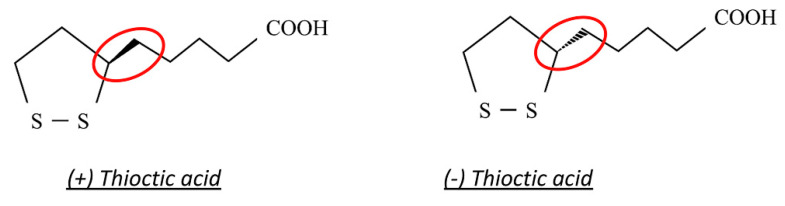
Chemical structure of (+)-thioctic acid and (−)-thioctic acid. Red circles represent the chiral center of the molecule.

TIO is a dithiol eight-carbon molecule (chemical formula: C_8_H_14_O_2_S_2_) [94]. It presents a chiral center inducing its isomerization into two optical enantiomers, (+)-TIO and (−)-TIO (Figure 5) [95]. The (+)-TIO is synthesized de novo in mammalian mitochondria by the enzyme lipoic acid synthase (LASY), from octanoic acid and cysteine [96]. Thanks to its amide linkage to a lysine residue, (+)-TIO acts as a cofactor for some critical mitochondrial enzymes, such as pyruvate dehydrogenase (PDH), branched-chain α-keto-acid dehydrogenase (KDH), and α-ketoglutarate dehydrogenase (KGDH) [97].

TIO has been widely studied since the 1950s when its antioxidant properties were first discovered [98]. It has been pointed out that TIO is effective in relieving some symptoms associated with certain diseases like diabetes, age-related cardiovascular and neuromuscular defects, antipsychotic drug-related weight gain, and metabolic obesity [99,100,101,102]. Its potential effects on different types of diseases have drawn attention since the results from studies were promising, namely in the field of neurodegenerative conditions [103]. Additionally, the number of clinical trials increased to deepen knowledge on other TIO therapeutic properties and found hopeful effects. TIO is absorbed from a wide variety of animals and vegetables, and once it is inside the cell, it starts to convert its reduced form, dihydrolipoic acid [104]. It appears that TIO or its reduced form dihydrolipoic acid (DHLA) possesses many biochemical functions, acting as biological antioxidants, as metal chelators, able to regenerate endogenous antioxidants, and as a modulator of the signaling transduction of several pathways [105]. Briefly, it also has a protective mechanism that protects membranes by interacting with vitamin C and glutathione (GSH), which may, in turn, recycle vitamin E. Moreover, dihydrolipoate may exert prooxidant actions through the reduction of iron. The application of TIO is beneficial in several oxidative stress models like ischemia-reperfusion injury, diabetes (both α-lipoic acid and dihydrolipoic acid exhibit hydrophobic binding to proteins which might prevent glycation reactions, such as albumin), neurodegeneration, cataract formation, and radiation injury [93].

In medicine, TIO has been shown to scale back symptoms of diabetic polyneuropathy, and several clinical trials established some efficacy and an excellent safety profile in this patient population [106]. Previous studies suggested that alpha-lipoic acid has anti-obesity properties [107]. In animal studies, it has been shown that TIO supplementation excites to reduce body weight and fat mass by decreasing food intake and enhancing energy expenditure, possibly by suppressing hypothalamic AMP-activated protein kinase (AMPK) activity [108]. However, studies in humans with TIO supplementation are limited, and therefore the results have been inconsistent. Some clinical trials suggest that TIO supplementation may help overweight or obese individuals [109,110], while other studies have observed no effects of TIO on weight [111,112]. Nevertheless, TIO appears to provide a large range of beneficial effects on obesity-related conditions such as insulin resistance, metabolic syndrome, and type 2 diabetes, including their complications such as vascular damage [106,113].

TIO has many clinically valuable properties [114]. It works as an enzymatic cofactor [93], and it is involved in glucose metabolism, lipid metabolism, and control gene transcription. TIO also restores the intrinsic antioxidant systems and supports their production or cell accessibility [94,95]. It efficiently removes heavy metals from the bloodstream. TIO is also responsible for oxidative stress [115]. The most identifiable characteristic of TIO over other antioxidant substances is that it reacts both as lipid and as water-soluble compounds [113]. TIO also has some other functions because it is involved in mitochondria-producing energy, acting as a cofactor for various enzymes that deal with metabolism [113].

Besides, TIO plays a key role in glucose metabolism. A racemic form of TIO was applied for the treatment of diabetic polyneuropathy-associated pain and paresthesia [116,117]. TIO also has a pivotal function in energy transduction through mitochondria [118,119]. Two reduced or oxidized thiol groups are present within TIO. It inactivates free radicals, and the reduced form interacts with ROS.

TIO is synthesized de novo at small amounts within the body from cysteine and fatty acids, and thus it is necessary to supplement it from exogenous sources [120]. TIO improves glycemic control [118], alleviates diabetes mellitus (DM) complications [121,122], and even symptoms of peripheral neuropathy, and at the same time, it effectively lessens the heavy metals toxicity [123].

TIO can enhance body weight and fat mass loss through its ability to suppress the hypothalamic region. AMPK and decreasing dietary energy intake reduce lipoprotein lipase activity, increase energy expenditure, lipolysis, insulin sensitivity, and inhibit lipogenesis [97,99,124,125,126]. Furthermore, clinical trials have demonstrated that TIO is safe, and no serious adverse effects have been reported [127,128]. Discrepancies in findings might be related to different study designs, such as characteristics of study samples, dosage, and duration of the studies.

### 2.4. Curcumin

**Figure 6:**
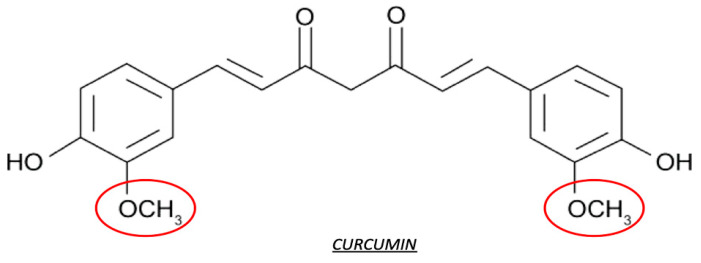
Configuration of curcumin. Red circles represent the reactive site of the molecule.

Curcumin is a bioactive polyphenol derived from spice turmeric. Curcumin plays several biological functions, such as antioxidative, anti-inflammatory, and anti-angiogenesis in different organs, including adipose tissue (Figure 6).

Curcumin contains approximately 70% carbohydrates, 13% moisture, 6% protein, 6% essential oils, 5% fat, 3% mineral (potassium, calcium, phosphorus, iron, and sodium), 3–5% curcuminoids, and tiny amounts of vitamins [129,130]. Curcumin was isolated as diferuloylmethane, or 1,6-heptadiene-3,5-dione-1,7-bis (4-hydroxy-3-methoxyphenyl)-(1E,6E) [131].

Curcumin, and other dietary polyphenols, due to their potential mechanisms of metabolic actions, may be useful for the therapy of obesity. Nowadays, the controversy concerning the therapeutic potential of curcumin indicates challenges and interest in this research field. Here, we try to introduce some investigations on antioxidant properties of curcumin that exhibit its application to metabolic diseases, in particular to obesity and diabetes. The beneficial effect of curcumin has been reported by some preclinical and clinical investigations which showed that curcumin and other dietary polyphenols reduce body weight, ameliorate insulin sensitivity, and prevent diabetes development both in rodent models and in prediabetic subjects [132]. We also found improvements in insulin actions by significantly decreased serum triglyceride levels after curcumin treatment and reducing the inflammatory cytokines IL-1β and interleukin 4 (IL-4) in the serum levels of obese individuals [133,134]. Recent studies suggest that there are some links to changes in gut microbiota because of the metabolic effects of curcumin and polyphenols. In this way, research into the supplementation of curcumin continues to provide novel insights into metabolic regulation and its effectiveness in reducing the oxidative stress burden in obese individuals.

**Figure 7:**
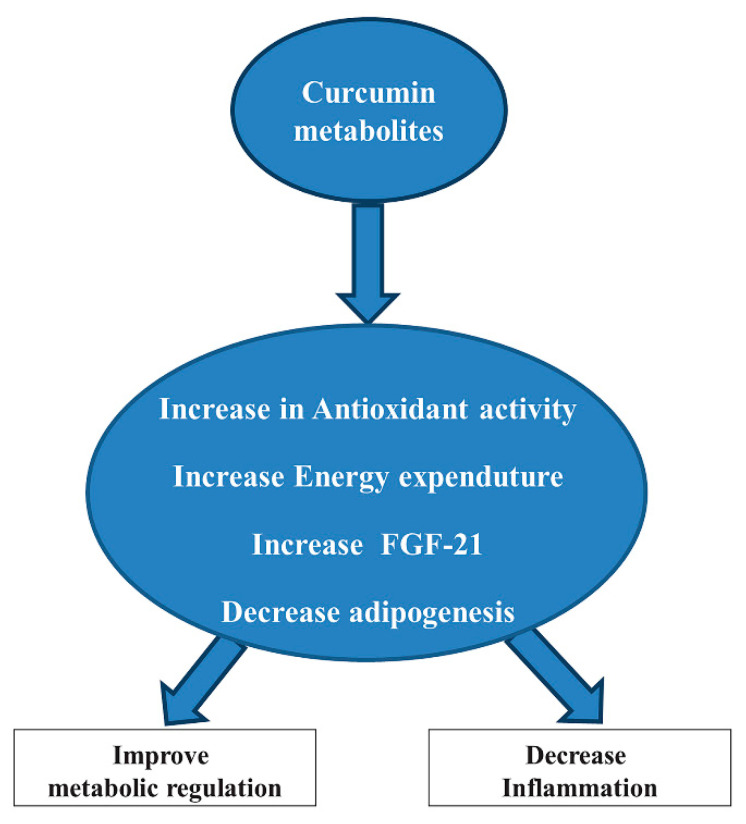
Possible antioxidant effects of curcumin metabolites. Abbreviation: Fibroblast growth factor 21 (FGF-21).

Curcumin may have a relevant effect on adipogenesis. Curcumin treatment suppressed the expression of adipogenic genes peroxisome proliferator-activated receptor γ (PPARγ) and CCAAT-enhancer-binding proteins α (C/EBPα) in primary human adipocytes and murine 3T3-L1 adipocytes [135]. Due to its antiadipogenic effects, curcumin suppresses the preadipocyte’s maturation, preventing their differentiation by inactivation of MAPK, including extracellular signal-regulated kinase (ERK), c-Jun N-terminal kinase (JNK), and p38, that inhibit 3T3-L1 adipocyte differentiation [136]. The inactivation effect by curcumin may also suppress the PPARγ expression in a dose-dependent manner in human adipocytes. Besides, curcumin also showed anti-inflammatory effects. In 3T3-L1 adipocytes, the secretion of a proinflammatory cytokine and the MCP-1 were inhibited by the pretreatment of curcumin [137]. Curcumin showed beneficial effects on energy metabolism and the reduction of body weight. Some evidence suggests that in rats, two weeks of high dietary curcumin supplementation reduced epididymal adipose tissue and increased fatty acid β-oxidation, indicating the increase of energy expenditure after curcumin treatment [138]. In rodent models of HFD that induced insulin resistance, many supplementations of compounds targeted to inhibit the pathogenic factors led to improvements in insulin sensitivity and resistance to weight gain. Among these compounds, curcumin has shown some beneficial effects. Nevertheless, the observation of decreased adipogenesis and/or fat mass accumulation in HFD animals, and the inhibition of adipocyte proliferation and differentiation by curcumin supplementation, suggest an enhanced catabolism in adipose tissue [139,140]. Moreover, another study demonstrated that curcumin promoted browning of WAT in diet-induced obese mice, which indicates increased energy expenditure as another mechanism linking curcumin supplementation to reduced fat mass deposition [141]. Several in vivo studies in rodents demonstrate anti-inflammatory effects of curcumin both in an HFD animal model of obesity and in genetic obesity (ob/ob mice) [132]. Moreover, curcumin, similarly to other polyphenols, could activate the nuclear factor erythroid 2–related factor 2/Kelch-like ECH-associated protein 1 (Nrf2-Keap1) antioxidant response due to the presence of a reactive Michael acceptor that reacts with cysteine residues on Keap1 [142]. This sulfhydryl reactivity may explain the other actions of curcumin but, at the same time, it has an apparent lack of specificity, particularly in cell culture experiments [132]. Curcumin also showed anti-inflammatory functions. In HFD-induced obesity and genetic obesity (ob/ob mice) models, curcumin reduced adipose tissue inflammation by reducing macrophage infiltration into adipose tissue and by increasing adiponectin production (Figure 7) [143,144].

Furthermore, other investigations suggested that high doses of curcumin can prompt gastrointestinal upset, skin inflammation [145], and advanced liver toxicity in humans [146]. Major pathogenetic factors which link obesity to metabolic diseases involve increasing levels of circulating free fatty acids, endoplasmic reticulum (ER) stress, altered levels of adipokines, decreased adiponectin, and infiltrating macrophage-derived cytokines, such as increased MCP-1, TNF-α, and IL-6 [147,148,149,150,151,152,153,154].

### 2.5. Caffeine and Catechin

Green tea is one of the most widely consumed beverages. Nowadays, its medicinal properties have been widely explored. The green tea plant named *Camellia sinensis* is a member of the Theaceae family, and green tea is produced from its leaves. *Camellia sinensis* thrive mainly in tropical and subtropical climates. The tea plant is cultured from seed which needs 7 to 10 years to become ready for harvesting. Green tea is a non-alcoholic beverage. Two different groups of tea have been found, particularly one is *Camellia sinensis. var*. *assamica*, used in India, and the other one is *Camellia sinensis var. sinensis,* used in Japan and China [155].

Green tea contains several bioactive components, including free polyphenols, amino acids, and caffeine. Chemically, caffeine is a methylxanthine (1,3,7-trimethylxanthine). Many plants contain caffeine in their seeds, fruits, and leaves. Besides coffee and tea, these plants include cacao beans, yerba matte leaves, and guarana berries [156]. Preclinical studies reported that caffeine had antioxidant properties, for example in brain and in liver. Chronic coffee and caffeine intake diminishes the lipid peroxidation and protects membranes from damage caused by ROS, and amplifies the activity of antioxidant enzymes (GSH and SOD) [157,158,159]. Caffeine also provided anti-fibrogenic and anti-inflammatory effects (decreased serum levels of cytokines TNF-α, IL-1β, and IL-6) that were associated with recovery of hepatic histological and functional alterations from thioacetamide-induced hepatotoxicity [159]. Experimental studies in animals reported a lower incidence of obesity, metabolic syndrome, and type 2 diabetes in regular coffee drinkers, of 3–4 cups per day [160,161]. Indeed, coffee as well as caffeine reduced lipogenesis, regulated lipid uptake and transport, increased fatty acid β-oxidation, increased lipolysis, and reduced lipid digestion, as reviewed in [161].

Basically, catechins are the major class of polyphenols that have high biological activity. Generally, catechin and its derivatives are expected in the green tea infusion, including epicatechin, epicatechin-3-gallate, epigallocatechin, and epigallocatechin-3-gallate (EGCG) [162]. Green tea is extracted from boiling fresh leaves at high temperatures. This procedure leaves the polyphenol content intact by inactivating the oxidizing enzymes. Flavanols or catechins are the most commonly found polyphenols in green tea.

There are several investigations about green tea polyphenols that have demonstrated significant antioxidant, anti-inflammatory, antibacterial, antiviral, and anti-angiogenic properties in numerous human, animal, and in vitro studies [163,164]. Green tea also has some beneficial effects against cancer, obesity, diabetes, and cardiovascular diseases [165].

Their antioxidant perspective is directly involved in the combination of aromatic rings and hydroxyl groups that make up their structure. It may be a result of the binding and neutralization of free radicals by the hydroxyl groups. Moreover, this kind of polyphenols augment the activity of hepatic detoxification enzymes, which promote the detoxification of xenobiotic compounds. These are also able of chelating metal ions such as iron that can generate ROS [166,167]. Green tea polyphenols have some adverse effects. They inhibit the production of arachidonic acid, metabolites such as pro-inflammatory prostaglandins and leukotrienes, and as a result, they decrease the inflammatory response.

Green tea has the ability to decrease the denaturation of proteins and increase the production of anti-inflammatory cytokines [168,169]. Besides, they can control the number of free radicals by binding to ROS, upregulating basal levels of antioxidant enzymes, and increasing the activity of these antioxidant enzymes [170,171,172].

**Figure 8:**
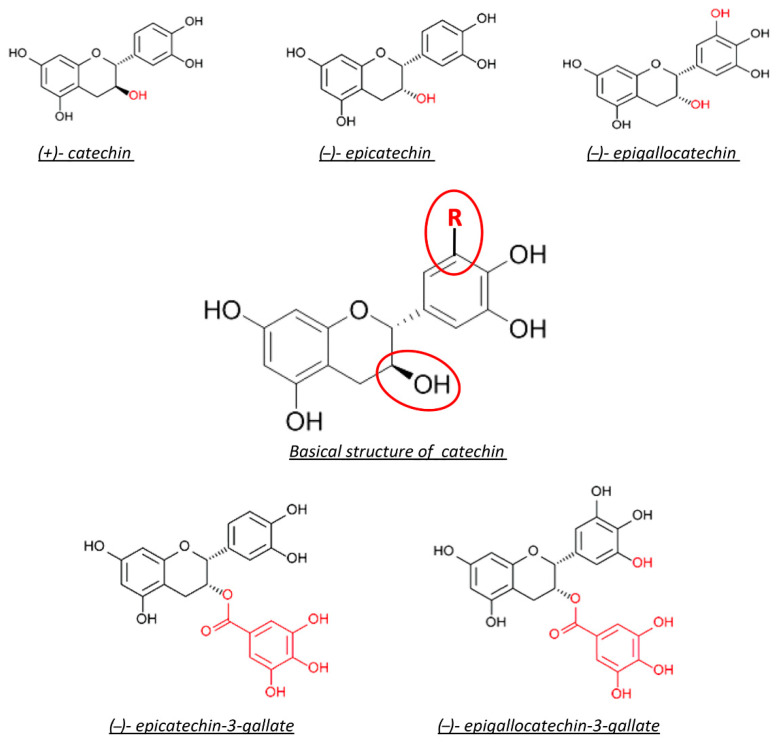
Chemical structure of green tea catechin. Red circles represent the reactive site of the molecule.

We mentioned before that there are mainly five catechins in green tea. They are (+)-catechin (C), (−)-epicatechin (EC), (−)-epigallocatechin (EGC), (−)-epicatechin-3-gallate (ECG), and (−)-epigallocatechin-3-gallate (EGCG) (Figure 8). Among these four, the most abundant catechin is EGCG (∼60%), and the next most abundant is EGC (∼20%), then ECG (∼14%) and EC (∼6%). EGCG is the most studied catechin, which is related to health benefits, although EGC and ECG have been studied as well. There is a variation in the number of catechins in any particular green tea beverage. However, standardized extracts are available for use as supplements [173,174].

Several studies on humans and animals have demonstrated the ability of EGCG’s to block the inflammatory response due to ultraviolet A and B radiation and significant inhibition of the migration of neutrophils, which occurs during the inflammatory process [175,176]. The thermogenic properties of green tea indicate a synergistic interaction between the caffeine content and catechin polyphenol that may be a result of prolonged stimulation of thermogenesis.

Thermogenesis is a bioenergetic process, which is associated with adiposity, obesity, insulin sensitivity, blood glucose concentration, and its related disorders [155]. The brown adipocytes have two thermogenic cells, which are highly expressed in uncoupling protein 1 (UCP1). In terms of thermogenesis, UCP1 is activated and evaporates energy as heat instead of ATP synthesis [177,178]. Several studies have shown that green tea and its components can induce fat oxidation. Catechins in green tea increase energy expenditure (EE) by inhibiting COMT, which degrades catecholamines, such as norepinephrine [179]. The inhibition results in the stimulation of catecholamines and the increase of EE.

EGCG’s catechins increased, directly or indirectly, total plasma antioxidant activity. Besides, they inhibited LDL oxidation and decreased high-density lipoprotein (HDL) cholesterol [180,181]. A randomized trial revealed that in postmenopausal women, total blood cholesterol was significantly reduced after the use of green tea extract for four weeks, compared to the control group [182].

Some studies are presenting that green tea is used for the prevention and treatment of obesity and type 2 diabetes. Green tea catechins decreased serum glucose levels [183], and especially EGCG could mimic the action of insulin and enhance insulin resistance [184,185], and catechins also reduced the absorption of triglycerides, cholesterol, and also regulated the glucose and lipid metabolism in a HFD mouse model [185,186,187]. The bioactive components of green tea had reduced miRNA levels of lipogenic, adipogenic, and fatty acid uptake genes. In green tea, EGCG is a bioactive molecule that reduces the expression of FAS and glycerol-3-phosphatase acyltransferase, acetyl coenzyme A carboxylase-1, and sterol-coenzyme A desaturase 1 (SCD1) mRNA [188,189].

## 3. Discussion

Although several drugs are approved for the treatment of obese patients, many of them were withdrawn due to severe adverse events such as heart and psychiatric disorders [190]. Recent studies demonstrated that consumption of bioactive components of food such as phenolic compounds is positively associated with reducing the risk of obesity and associated chronic diseases. [191,192]. Thus, creating new dietary treatments based on various bioactive components in food has been emerging as a new possible intervention against obesity [193].

As summarized in Table 1, different antioxidant compounds could have possible anti-obesogenic and anti-inflammatory effects.

Other compounds demonstrated positive effects in animal models of obesity. Some studies have suggested that ursolic acid may reduce fat storage by enhancing lipolysis in adipocytes and inhibiting pancreatic lipases [201], decreasing the synthase of fatty acid activity [202], and acting as a PPARα agonist to regulate hepatic lipid metabolism [203]. Furthermore, ursolic acid may increase energy reserves in muscles by enhancing glycogen storage and lean muscle mass through increased sensitivity to insulin and insulin-like growth factor 1 (IGF-1) [204].

Vitamins C and E are antioxidants and also act as cofactors in many enzymatic reactions. To prevent the oxidation of membrane lipids, vitamin E acts as a peroxyl scavenger [205]. Vitamin E is present in low amounts, and it recycles oxidized form to the reduced form, and it is coupled with vitamin C, which is present in the body at a greater concentration. Vitamin C is a water-soluble vitamin naturally present in some foods that represent an essential dietary component for animals and humans [206,207].

Zinc acts on several antioxidant enzymes as a cofactor, such as copper-zinc (Cu-Zn) SOD [208]. There have been supplementations of Zn tested to view the effects on oxidative stress and type 2 diabetes. Zn supplementation showed an improved glycemic control and lipid profile in patients with higher postprandial glucose levels or higher fasting blood glucose [209].

Omega-3 polyunsaturated fatty acids (Omega-3 PUFAs) have been shown to contribute to human obesity [210]. Omega-3 fatty acids are dietary essentials of PUFAs [211]. The vegetal form of Omega-3 fatty acids is a short-chain fatty acid α-linolenic acid obtained from plant oil, including leafy vegetables, walnuts, soybean oil, canola oil, and flaxseed oil [212]. The marine forms of Omega-3 fatty acids are the long-chain fatty acids, such as docosahexaenoic acid (DHA) and eicosapentaenoic acid (EPA). They are obtained from seafood, fish, and algae [213]. Obesity has been connected with low levels of Omega-3 PUFAs [214]. In fact, the supplementation of Omega-3 PUFAs may help to reduce the incidence of obesity and its comorbidities [215].

Coenzyme Q10 is involved in energy production via the mitochondrial electron transport chain [216]. Some literature reviews suggested that coenzyme Q10 has anti-inflammatory effects in vitro, but coenzyme Q10 supplementation seems to have only an antihypertensive effect, and there have no benefits to reduce the body weight, fat mass, or glycemia [217].

## 4. Conclusions

Obesity is a medical condition consisting of abnormal deposition of adipose tissue, with a negative effect on health status. An imbalance between caloric intake and energy expenditure results in fat accumulation due to excessive lipogenesis in adipose tissues. Many preclinical and clinical studies suggested a possible convergence of an inflammatory state, which results in chronic inflammation and oxidative stress that is localized within adipose tissue. Oxidative stress and inflammation play a crucial role in developing obesity-related metabolic complications. No specific therapeutical policies are available to counteract these complications. The evidence of the possible protective properties of natural antioxidant compounds, despite no specific effects shown on weight gain, could represent an important strategy to prevent metabolic alterations in adipose tissue. More specific clinical trials are necessary to confirm the role of these compounds on human health.

## Figures and Tables

**Table 1:** Effects of dietary antioxidants on inflammation and obesity (in vivo and in vitro studies).

Antioxidant	Sources	Bioactive Dose of Antioxidant	Moderator	Metabolic Marker	Inflamatory Marker	Reference
**Anthocyanine**	Tart cherry powder	Cyanidin(3-sophoroside), cyanidin (3-glucosylrutinoside), cyanidin- glucose(3-glucoside) insulin, cyanidincholesterol (3-rutinoside), triglyceride (TG) peonidin (3-glucoside).	Zucker rats	Reduced glycemia and insulinemia as well as improved insulin resistance.	Decreased plasma levels of IL-6 and TNF-α.	[194]
	Whole blueberry powder	Delphinidins, cyanidins, peonidins, malvidins.	C57BL/6 mice	Reduced levels of fasting glucose improved insulin tolerance test (ITT)	Reduced TNF-α expression in adipose tissue.	[195]
	Blueberry juice	Cyanidi(3-galactoside) cyanidi (3-arabinoside delphinidi(3-glucoside), delphinidinadiponectinpetunidin(3 arabinoside), malvidin (3-galactoside), malvidin(3-glucoside). Dose: 4.09 mg/mL	Mice	Reduced body weight, decreased the level of TG, leptin, and cholesterol, percentage of WAT.	Reduced TNF-α and IL-6 expression.	[196]
	Purple sweet potato	Cyanidnin(3-caffeylferulysophoroside-5-glucoside), peonidin(3caffeylferulysophoroside-5-glucoside). Dose: 4.28 to 12.84 µg/mL	Murine 3T3-L1 adipocytes	Decreased leptin and adipogenic factors.	Decreased COX-2, MCP-1, IL-6.	[197]
**Resveratrol**	Red wine, acai, blueberry, cranberry, pomegranate, Japanese knotwood, Ziziphus.	Resveratrol-4′-*O*-glucuronide, resveratrol-3-*O*-glucuronide and resveratrol-3-*O*-sulfate. Dose: 15 mg/kg body weight/day (gavage).	Male Zucker rats	Decreased TG content, increased epinephrine-stimulated glycerol release, increaseD hormone-sensitive lipase (HSL) mRNA.	Reduced IL-6, TNF-α, IL-1β, and NF-κB.	[60,198]
			Obese Zucker (fa/fa) rats			
		1, 10, 25 µM resveratrol, resveratrol-4′-*O*-glucuronide, resveratrol-3-*O* glucuronide and resveratrol-3-*O* sulfate.	Murine 3T3-L1 adipocytes	Increased SIRT1 mRNA, increased TG content, increased peroxisome proliferator-activated receptor gamma coactivator 1-alpha (PGC-1α) mRNA, increased adipose triglyceride lipase (ATGL) mRNA, increased HSL mRNA.	Reduced IL-6 and TNF-α.	[199]
**Lipoic acid**	Red meat, spinach, broccoli, tomatoes, peas, Brussels sprouts.	Oxoaciddehydrogenase, pyruvatedehydrogenase complex, 2-oxoglutarate dehydrogenase complex.	HFD-induced obesity	Improved glycemic control and lipid profile, decreased weight.	Reduced IL-6 and TNF-α.	[200]
**Curcumine**	Rhizome, or rootstalk of the turmeric plant.	1,7-bis(4-hydroxy-3-methoxyphenyl)-1,6-heptadiene-3,5-dione.	Primary human adipocytes and murine 3T3-L1 adipocytes	Suppressed the expression of adipogenic genes, PPARγ, and C/EBP α.	Reduced (MCP-1, a proinflammatory cytokine.	[137]
		1,7-bis(4-hydroxy-3-methoxyphenyl)-1,6-heptadiene-3,5-dione	HFD-induced obesity and in genetic obesity (ob/ob mice).	Reduced body weight and energy metabolism, reduced epididymal adipose tissue, increased fatty acid β-oxidation.	Increased adiponectin production and reduced inflammation.	[144]
**Catechin**	*Camellia sinensis* leaves and buds, green tea.	(−)-EGCG	Obese Zucker (fa/fa) rats	Reduced deleterious effects, including hepatic injury.	Decreased TNF-α, IL-1β, COX-2, and matrix metallopeptidase 9 (MMP-9).	[165]
**Caffeine**	Coffee and tea.	1,3,7-trimethylxanthine. Doses: 20 and 40 mg/kg per day; 37.5 mg/kg per day; 3–4 cups of coffee per day.	Male rats	Reduced lipogenesis, regulated lipid uptake and transport, increased fatty acid β-oxidation, increased lipolysis and reduced lipid digestion. Decreased lipid peroxidation and increased antioxidant enzyme activities.	Decreased serum levels of inflammatory cytokines TNF-α, IL-1β, and IL-6.	[157,158,159,160,161]
